# Instant snapshot of the internal structure of Unzen lava dome, Japan with airborne muography

**DOI:** 10.1038/srep39741

**Published:** 2016-12-23

**Authors:** Hiroyuki K. M. Tanaka

**Affiliations:** 1 Earthquake Research Institute, The University of Tokyo, 1-1-1 Yayoi, Bunkyo, Tokyo 113-0032, Japan

## Abstract

An emerging elementary particle imaging technique called muography has increasingly been used to resolve the internal structures of volcanoes with a spatial resolution of less than 100 m. However, land-based muography requires several days at least to acquire satisfactory image contrast and thus, it has not been a practical tool to diagnose the erupting volcano in a real time manner. To address this issue, airborne muography was implemented for the first time, targeting Heisei-Shinzan lava dome of Unzen volcano, Japan. Obtained in 2.5 hours, the resultant image clearly showed the density contrast inside the dome, which is essential information to predict the magnitude of the dome collapse. Since airborne muography is not restricted by topographic conditions for apparatus placements, we anticipate that the technique is applicable to creating images of this type of lava dome evolution from various angles in real time.

Lava dome eruptions are one of the most hazardous volcanic eruption types. For example, gravitational collapses of Heisei-Shinzan lava dome of Unzen volcano in 1989 caused repeated pyroclastic flows, resulting in the evacuation of more than 10,000 local residents. However, the internal structure of a lava dome, particularly the extent of the surrounding low-density talus has been an unknown factor although it is likely critical to prediction of its collapse magnitude^1^. The lava dome generally consists of two units: a relatively soft interior (core) and a solidified outer region (talus). The outer region is less cohesive and breaks apart generating debris. This debris builds up the low-density talus that eventually surrounds the dome with a broad conical form^2^. A dome eruption is first dominated by the extrusion of dense, degassed, and high viscosity magma plugs called spines^3^. As the eruption progresses, lava extrudes into the core^2^, and a transition zone is generated between the core and the talus.

Lava dome collapses can occur either within active domes or within magma stored at high levels on the volcanic edifice^4^. It can be triggered by various events, for example, heavy rainfall^5^, seismicity^6^, and pressurization^7^. Carn *et al*.^8^ reported that the pyroclastic flows were observed during an intense rainfall event in Unzen, Japan, Merapi, Indonesia, Santiaguito, Guatemala and Mt. Saint Helens, USA, and it was interpreted that they were all caused by the erosion of the talus^1^. However, they did not always result in catastrophic events^8^. It is thought that the thickness of the talus is one of the key factors, which controls the collapse magnitude since erosion of the talus may expose the hot and dense core, leading to an explosive event^1^. For example, Saint Helens lava dome was observed to be surrounded by a thick layer of talus in early December 1999^2^. This is probably the reason why the November 1999 and December 2000 Mt. Saint Helens collapses were not major collapses^8^. Visualization of the internal structure of growing lava domes within a few hours would provide a snapshot of the talus thickness and therefore, repeated measurements would provide direct information on its evolution that might be essential to facilitate risk mitigation of dome collapses.

Muography produces a projection image (muogram) of a gigantic body by mapping out the number of muons that are transmitted through it. This technique has been applied to explore the internal structures of volcanoes^9^,^10^,^11^,^12^,^13^,^14^,^15^, faults^16^,^17^, caves^18^,^19^,^20^, carbon capture and storage (CCS)^21^,^22^, industrial plants^23^, and historic architecture^24^. The flux of open-sky vertical muons is ~70 m^−2^ s^−1^ sr^−1^ (ref. 25) and this flux is reduced according to the relationship described by cos^2^θ^*^, where θ^*^ is the muon’s arriving angle from zenith. The muon flux is also reduced after passing though matter. The resultant image quality depends on the number of surviving muon events that are acquired within the given observation period. Conventional land-based muographic measurements have required a measurement time of at least several days^26^ and sometimes longer^9^,^10^,^11^,^12^,^13^,^14^ to image the internal structure of a volcano. This is mainly due to strong attenuation of the muon flux after passing through a long distance of rock. Since the required exposure time is anti-proportional to the detector’s active area, enlarging the active areas, ranging from 0.5 to 1 m^2^ (refs 9, 10, 11, 12, 13, 14) helped to reduce exposure time. For example, Tanaka *et al*.^26^ utilized a detector with an active area of 2 m^2^ to achieve a time resolution of 3 days for the Satsuma-Iwojima measurement in 2013. This apparatus weighed more than 10 tons including a radiation shield and, required more than 5 × 5 × 3 m^3^ for apparatus housing and a stable electricity source; therefore, practical implementation would be challenging for some field conditions.

Airborne muography is another solution to drastically shorten the exposure time of the measurement. In airborne muography, the muon trackers are equipped inside the aircraft that flies and hovers near the target of interest to record muons (Supplementary Movie 1). The data are analyzed in the aircraft in real time to produce a muographic image of the target. Airborne muography has three major advantages over land-based muography: (A) electricity availability from the power generator equipped in the aircraft; (B) fast transportation and installation of the detector; and (C) the detector is in the optimum position for collection of muon events. Condition (C) in particular shortens the time required for obtaining images. By getting the detector as close to the target of interest as possible, the viewing solid angle (Ω) of the region of interest is maximized. The value of Ω increases as a function of the distance from the target, and as a consequence, the recorded number of muons that pass through the region is also increased. This is one of the most effective techniques to shorten the time required for collecting muons after they have passed through the target region. For example, if the distance to the target is reduced to a half of the original value, it will take a quarter of the original time to produce the same quality images. In this work, the first airborne muography measurement was conducted and as a result, the internal structure of Heisei-Shinzan lava dome of Unzen volcano, Japan was imaged in 2.5 hours. The aircraft hovered at an altitude of 1350 m and a horizontal distance of a few meters from the steep surface of the Heisei-Shinzan lava dome. In the following sections, the apparatus and technique used for the present measurement and its results will be reported and discussed. The density contrast inside the lava dome was captured with muography at the fastest speed ever reached.

## Results

### Modeling

**Figure 1:**
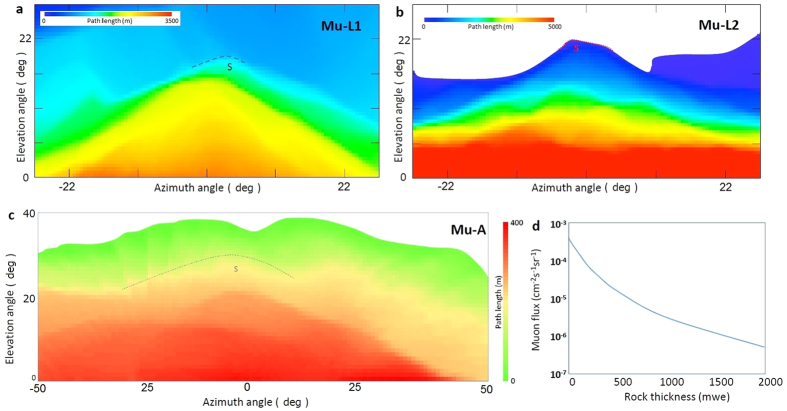
Topographic condition of Unzen volcano. The path length distributions calculated for Heisei-Shinzan lava dome from the observation points Mu-L1 (**a**), Mu-L2 (**b**), and Mu-A (**c**) are plotted as a function of azimuth and elevation angles. The dotted lines labeled with S indicate the location of the 1994 spine. (**d**) The transmitted muon flux at an elevation angle range of 24 ± 12° is plotted as a function of the rock thickness in units of m water equivalent.

For land-based muography, two observation points (Mu-L1 and Mu-L2) were selected based on accessibility and infrastructures. From Mu-L1, a mountain between the MOS and Heisei-Shinzan lava dome (HS) adds 1 km of solid rock to the muon’s path length. The path length distributions calculated from Mu-L1 and Mu-L2 are plotted in Fig. 1a,b, respectively. On the other hand, from Mu-L2, there are no obstacles between the MOS and HS. However, the distance to HS is farther than Mu-L1. The distance between the peak of HS and Mu-L1 and Mu-L2 is 1930 m and 2370 m, respectively. Therefore, the diameter of the peak region of HS of roughly 150 m corresponds to a viewing angle of ~4.6° and ~3.4° from Mu-L1 and Mu-L2, respectively.

On the contrary, the observation points for airborne muography are more flexible. An observation point (Mu-A) was selected for the present measurement. The distance between the peak of HS and Mu-A is ~200 m and the path length distribution calculated from Mu-A is plotted in Fig. 1c. The rock thickness of the HS peak measures ~200 m. Figure 1d shows the near horizontal (an elevation angle range of 24 ± 12°) muon flux as a function of rock thickness. By employing the gravimetrically obtained reference bulk density value (0.7 gcm^−3^)^13^ for estimating the number of muons after passing through HS, a flux of 7.2 × 10^−6^ cm^−2^ s^−1^ sr^−1^ would be expected at Mu-L1 and, 1.2 × 10^−4^ cm^−2^ s^−1^ sr^−1^ would be expected at Mu-L2 and Mu-A. In order to resolve HS with a resolution of 100 × 100 m^2^, the viewing solid angle (*Ω*) from Mu-L1, Mu-L2 and Mu-A would be 2.7 millisteradian (msr), 1.8 msr, and 200 msr, respectively and thus, the number of expected muons would be ~2 × 10^−8^ cm^−2^ s^−1^, ~2 × 10^−7^ cm^−2^ s^−1^, and ~3 × 10^−5^ cm^−2^ s^−1^ at Mu-L1, Mu-L2 and Mu-A, respectively. The measurement at Mu-A was therefore expected to shorten the time required for imaging the peak region of HS by more than 2 orders of magnitude in comparison to the other 2 land-based measurements.

### Observation

**Figure 2:**
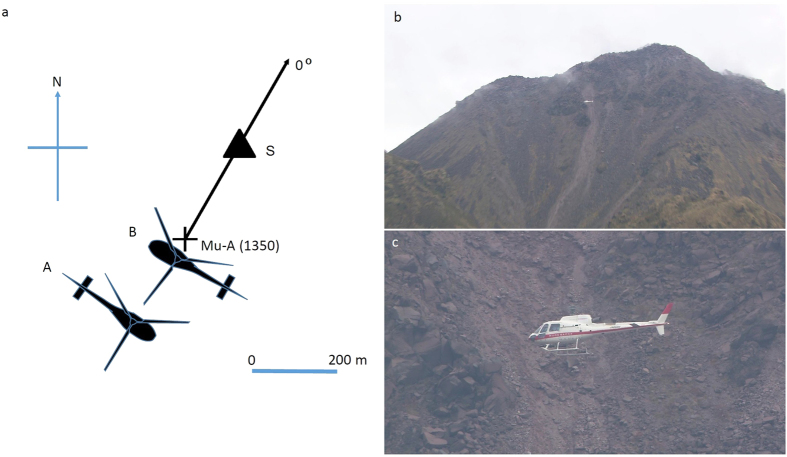
Scheme of the airborne muography measurement of Heisei-Shinzan lava dome, Japan. (**a**) The cross mark (Mu-A) indicates the hovering location. The triangle (S) indicates the 1994 spine. The aircraft was first directed toward the South-West direction (A) then flipped to be directed toward the North-West direction (B). The number indicates the elevation above sea level. The muon tracker was positioned toward the peak of the 1994 spine. (**b**) A photograph of the airborne muography measurement taken from the visual observation point. (**c**) Closer view of the measurement.

An earthquake swarm initiated the subsequent recurrent volcanic activities of Unzen volcano in 1989 and, HS was generated during this process^27^. The eruption was characterized by the incremental extrusion of spines (dense, degassed, and high viscosity magma plugs)^8^, followed by a series of viscous, dacitic lava lobes^27^. The lava dome growth continued until 1991 when the structure became unstable and then collapsed. At the end of the eruption, the spine (1994 spine) penetrated through the generated lava dome and its growth occurred within the last 5 months of the eruption, beginning in mid-October 1994 at an average rate of 0.8 m day^−1^ (refs 3,27). Spine extrusion proceeded obliquely at an inclination of 45 degrees toward the east-south-east^28^. The geometrical configuration of the present observation is shown in Fig. 2a.

**Figure 3:**
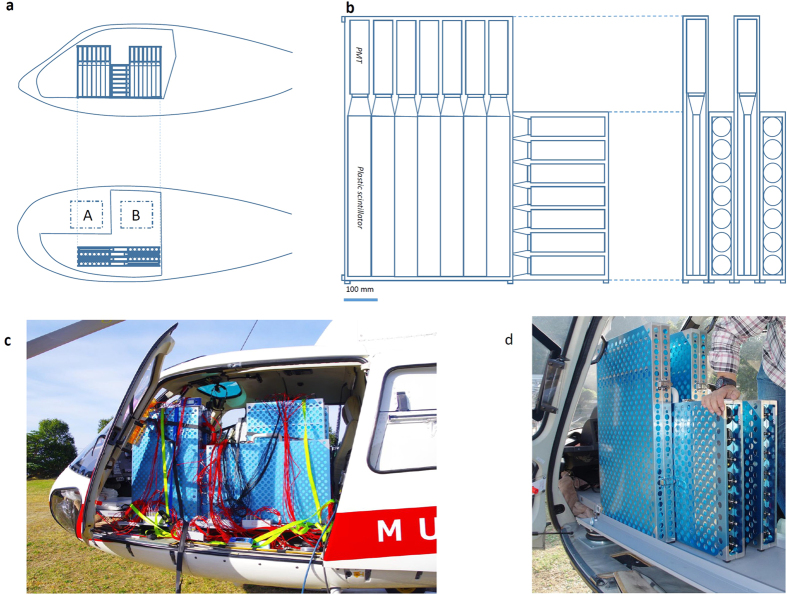
Experimental setup of the present airborne muography measurement. (**a**) The side (upper panel) and top (lower panel) view of the tracker arrangement. The dashed boxes show the locations of the pilot (A) and operator (B). (**b**) The schematics of the muon tracker. Each plastic scintillator is connected to the photomultiplier tube (PMT). (**c**) A photograph of the parallel muography observation system installed into the aircraft. (**d**) A closer view of the tracker prior to attaching cables.

Figure 2 shows the observational configuration of the present measurement of HS. The aircraft (Airbus AS350B3) hovered at a position of 200 m southwest from the peak of the 1994 spine at an altitude of 1350 m above sea level (asl) (Supplementary Movie 2). The parallel muon observation system (P-MOS) was installed on the port side of the aircraft as shown in Fig. 3a. The total active area and weight of the P-MOS was ~0.5 m^2^ and 120 kg, respectively. Including a pilot and operator, the total weight was 240 kg, which was less than the payload of the aircraft (400 kg). Since the weight of the P-MOS was roughly equal to the total weight of the pilot and the operator on the starboard side (Fig. 3a), an adjustment of the aircraft’s center of gravity was not necessary.

Background events enhance the apparent muon transmission rate; hence these false events tend to erroneously lower the apparent density along the muon path. Tanaka *et al*.^26^ reported that a lead radiation shield was effective for background event reduction in volcano muography. However, a radiation shield would add to the total weight of the P-MOS, which would likely exceed the payload of the aircraft. The radiation shield was therefore not used in the present measurement. Carbone *et al*.^13^ modeled that the background (BG) rate in Etna volcano was ~10^−5^ sr^−1^ s^−1^ cm^−2^ (~3000 m asl) for a similar kind of a segmented muon tracker without a radiation shield. In the present measurement, the expected muon transmission rate of 1.2 × 10^−4^ cm^−2^ s^−1^ sr^−1^ would be one order of magnitude higher than this BG rate. Furthermore, their measurement altitude was much higher than ours (1350 m asl) and thus, a much lower background rate than ~10^−5^ sr^−1^ s^−1^ cm^−2^ was expected in our measurement.

The P-MOS consisted of two compact MOSs. Each MOS consisted of a muon tracker (consisting of 2 segmented detectors as shown in Fig. 3b), high voltage (HV) supply, field-programmable-gate-array (FPGA)-based electronics for signal processing^29^, and data acquisition (DAQ) personal computer (PC). Each scintillation detector consisted of a plastic scintillator bar (ELJEN EJ-200), photomultiplier tube (PMT; Hamamatsu R7724), and acryl light guide. The dimension of the plastic scintillator bar used for this measurement was 50 cm long, 7 cm wide, and 1.8 cm thick and each scintillation detector weighed 1.2 kg. An array of 14 scintillator bars was used to form a segmented detector with an active area of 49 × 49 cm^2^. Since the distance between these segmented detectors was 16 cm, the tracking resolution was 24 ± 12°. This angular resolution corresponded to a spatial resolution of ~90 m at the peak of the 1994 spine. 28 signal outputs from each muon tracker were fed to the electronics to generate a histogram of the number of muon events as a function of azimuth and elevation angles^29^. The histogram was stored in a flash memory drive associated with the FPGA so that the DAQ PC could access the FPGA and download the data every minute. The total power consumption of the parallel MOS was 60 W (50 W and 10 W from PMTs and electronics, respectively) and, the electric power required for operating the parallel MOS was supplied by the aircraft’s power generator (400 W at the maximum).

The aircraft was first directed toward the southeast direction and collected muon data for 76 minutes (Session A) and then flipped toward the northwest direction and collected muon data for 81 minutes (Session B). The total observation period was 157 minutes. Since there were no obstacles on the opposite side of the lava dome (Fig. 1a), the open-sky muon data were recorded at the same time. The open-sky and transmitted muon counts recorded in Sessions A and B were respectively normalized to 60 minutes and were added. In the course of this process, the port-side and starboard-side data collected in Sessions A and B were combined to derive the total transmitted muon counts and, data collected in Sessions B and A were combined to derive the total open-sky reference muon counts. The results are shown in Tables 1 and 2. Muon transmission rates were subsequently calculated by dividing the transmitted muon counts by the open-sky muon counts (Table 3). Therefore, the geometrical acceptance and channel-dependent detection efficiency of each muon tracker were both canceled.

**Figure 4:**
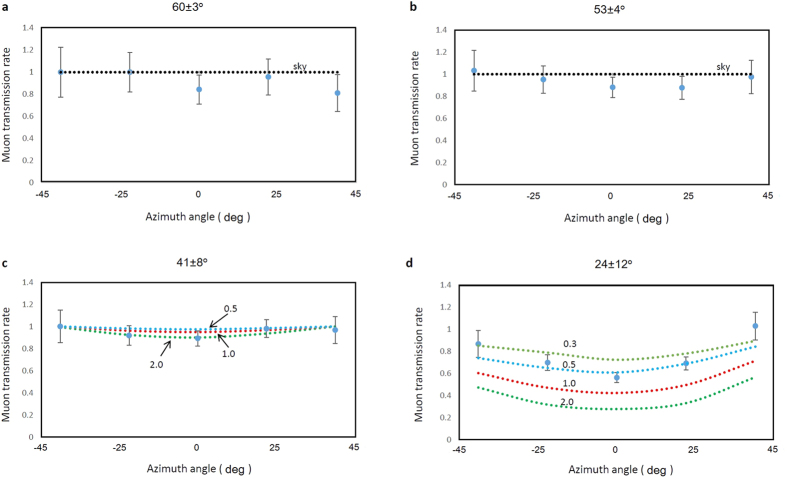
Muon transmission rate as a function of azimuth angle. The values are plotted for different elevation angle ranges ((**a**) 60 ± 3°, (**b**) 53 ± 4°, (**c**) 41 ± 8°, and (**d**) 24 ± 12°). Error bars represent 1 s.d. of statistical uncertainty (1 s.e.). Theoretical transmission rates (dotted lines) are also plotted by assuming various uniform densities of the lava dome. The numbers indicate the assumed density values.

**Figure 5:**
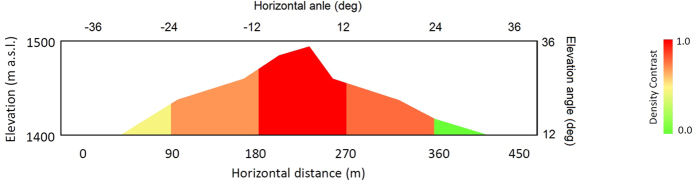
Density contrast of Heisei-Shinzan lava dome.

Each theoretical muon transmission rate was calculated based on the path length distribution of HS (Fig. 1d) and compared with the experimental muon transmission rate to derive the density contrast of the dome. In Fig. 4, the experimental muon transmission rate is compared with the theoretical rate derived by assuming uniform density values (0.3, 0.5, 1, and 2 gcm^−3^) for different elevation angle ranges (60 ± 3°, 53 ± 4°, 41 ± 8°, and 24 ± 12°). These angle ranges correspond to Fig. 5 shows the density contrast mapping of Heisei-Shinzan lava dome. Density values in this figure are normalized to the highest value. The overall average density of the HS dome above 1400 m asl was 0.50 ± 0.07 gcm^−3^. Density deviation measured for the center of HS (0.62 ± 0.12 gcm^−3^) from the average density excluding the central part (0.38 ± 0.08 gcm^−3^) was statistically significant.

## Discussion

### Density Contrast

The derived bulk densities (0.4–0.6 g cm^−3^) are close to the value (0.7 g cm^−3^) modeled by Carbone *et al*.^13^ for one of the summit cones of Etna volcano. Carbone *et al*.^13^ speculated that this low value was the result of (a) the highly vesiculated rocks or (b) low packing density following the depositional processes. The observed density (~0.6 g cm^−3^) at the central part of HS can be explained as a mixture of solid materials and brecciated deposits. By assuming that the average density (~0.4 g cm^−3^), excluding the central part, came from the brecciated deposits and by considering the final dimensions of the spine to be 150 m from East to West, 30 m from North to South, and 60 m high^27^, the bulk density of the solid materials (located above 1400 m asl) was derived to be ~2 g cm^−3^, which was consistent with the density of non-porous volcanic rock.

### Dome collapse assessment

The airborne muography technique offers an opportunity to obtain a more detailed image of the structure of a lava dome during its growth period. The obtained muographic image shows that the high-density solid materials (core) are situated inside the low-density brecciated deposits. Hale’s numerical modeling demonstrated that a low core volume fraction was expected in the latter phase of the dome formation once the extruded magma had had time to release its volatiles and to crystalize substantially.

A large core volume fraction is, on the other hand, expected during a dome growth period and is more likely to expose the core region of the dome. For example, large magnitude collapses were observed from 1999–2003 (Phase 2) and from 2005–2007 (Phase 3) at Mt. Saint Helens when the lava was crystal-poor. This phenomenon has been also seen in Soufrier Hills, Montserrat^1^. The time-dependent variations in the core volume fraction can be monitored during the dome formation and growth with airborne muography. The present work describes an incremental improvement to an existing technique. While the data collection is more rapid, the resolution of the data might be insufficient to go beyond a first-order model of a growing lava dome. However, in the future, the image resolution of muography will be improved by using, for example, the micromesh technology that provides up to a 100-nm resolution for incoming muon positioning^30^, and with this technology airborne muography would change hazard-management at a growing dome.

### 3-dimensional computational axial tomography

Tanaka *et al*.^31^ installed two muon trackers on the north and the east sides of Asama volcano, Japan to reconstruct the 3-dimensional (3d) structure of the volcano. However, in order to converge the inversion problem, bidirectional muography requires empirical information, such as the location and the size of the low-density region. Although low-density regions are likely to be located near the crater region, volcanoes generally have asymmetric structures and thus, the resultant images are distorted. This situation will not be drastically improved even if one or two more observation points are added since the number of images is still far below what is required (128–256 images) to reconstruct a high quality image that would be comparable to the quality of a medical computational axial tomography (CAT) image. The realistic number of observation points is generally restricted by accessibility and infrastructure availability (e.g. electricity) in land-based muography. Airborne muography removes this restriction. The present work confirmed that one muographic image of the Heisei-Shinzan scale lava dome could be taken in 2.5 hours. This means that in one day, 10 images of the lava dome could be obtained from 10 different directions.

## Methods

### Airborne muon detection

Muographic observations taken from within an aircraft remove topographic and infrastructural restrictions. The statistical fluctuation in the resultant image can be derived by taking the square root of the total number of muon counts after successfully penetrating the target of interest, which is proportional to the measurement time (*T*) and viewing solid angle (*Ω*) of the target. This viewing solid angle can be described by the following equation:


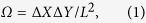


where Δ*X* and Δ*Y* are respectively the vertical and horizontal size of the target and *L* is the distance between the tracker and the target. Therefore, for the fixed Δ*X* and Δ*Y*, the number of collected muons increases as *L* decreases.

Another advantage of airborne muography minimizes the rock thickness to look through the target volume. The topography around a volcano is usually complicated (Fig. 1a) and it is generally difficult to get a clear pathway from the target volcano to the land-based detector. As can be seen in Fig. 1e, if the rock thickness is reduced to a half of the original value, the transmitted muon flux will be 10 times higher. Additional rock contributes to not only the reduction of the transmitted muons but also to the degrading of the density contrast; hence degrading the resultant images.

Airborne muography also removes the environmental restrictions associated with installation of the apparatus. Placement of the muon trackers at a steep flank covered with volcanic ash and loose rock is laborious and time consuming, and usually there is not enough space to anchor a large particle detector to the volcano surface. With the exception of film muography that utilizes the technique of nuclear emulsions^9^,^10^, muography measurements require electric power supplied by either commercial power or solar panels. However, it is unlikely that an infrastructural solution for electricity will be feasible near the peak of the most volcanoes.

### Positional control of the aircraft

**Figure 6:**
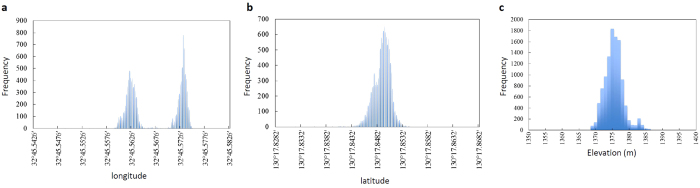
Distribution of the aircraft’s position recorded during the operation. The longitudinal (**a**), latitudinal (**b**) and elevation (**c**) distributions are shown.

During airborne muography, control of the aircraft position is important to reduce the blurring effect in the resultant image. A global positioning system (GPS; Akasakatec Poke-G) recorded longitude, latitude, and elevation information during the present measurement (Fig. 6). Since the spatial distance of an angle of 1 minute at 32°N is equivalent to 1.6 km, the GPS data showed that the fluctuation in positioning the aircraft was suppressed within ±3 m in each session. A bimodal distribution is shown in Fig. 6a. This reflects the fact that the aircraft could not return to exactly the same position of the Session A during Session B after refueling at the heliport. This discrepancy of ~15 m was much smaller than the positional resolution of the muon tracker at the HS peak (90 m). The yawing and pitching angles were also monitored by using digital levels, which confirmed that they were negligible in comparison to the angular resolution of 24 ± 12° during the operation. The location of the aircraft was also monitored visually from a distance (Supplementary Movie 2). This visual observation point (Nitta-Toge-Daini-Tenbosho (NTDT)) was located at the closest point to the peak of HS (3 km south-south-west (SSW) from the peak of HS) with space for the observation setup and a clear view to both Mu-A and HS. An image of the aircraft was magnified (100x) with a zoom lens (CANON HJ40) (40x) and its extender (2.5x) and the aircraft’s motion was recorded with a high-definition (1920 × 1080 pixels) camera (SONY HDW 750) at a frame rate of 60 frames per second (FPS).

### Data acquisition

**Figure 7:**
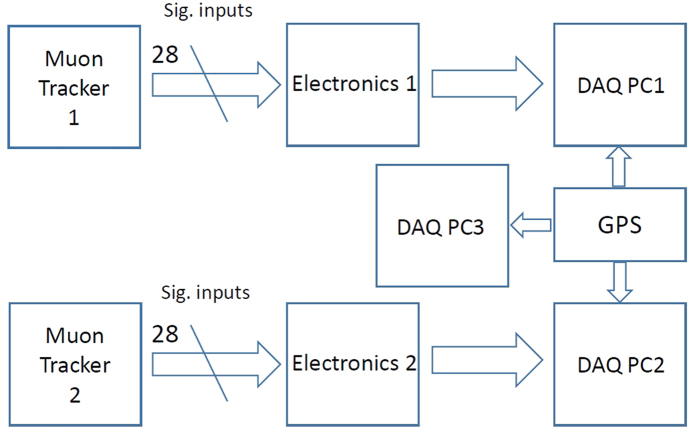
Block diagram of data processing of the current airborne muography measurement.

The clocks of the data acquisition personal computers (DAQ PCs) were all synchronized to the battery-operated global positioning system (GPS; Akasakatec Poke-G). The parallel MOS was operated by 3 DAQ PCs. Two of which (PC1 and PC2) were used for downloading the histogram data from the electronics that was associated with each tracker and, one of which (PC3) was used for recording the positioning information of the apparatus (Fig. 7). The elevation, longitude, latitude, and time data were saved in PC3 every second. In general, a GPS’s absolute positioning accuracy ranges in the order of tens of meters and therefore, only relative information was used. The aircraft was initially positioned visually by comparing the actual topography with the Geospatial Information Authority of Japan, Ministry of Land, Infrastructure, Transport and Tourism (GSI)’s topographic map and, the GPS was used to confirm any deviation from the initial position.

### Analysis

In order to analyze the muographic data, (A) the open-air muon energy spectrum, (B) continuous slowing down approximation (CSDA) range, and (C) HS topographic information were used. The spectrum measured by the KIEL-DESY collaboration^32^ was employed for the open-air muon energy spectrum. The shape of the spectrum was assumed to be invariant within an angle range between 24 ± 12°, but the integrated flux was adjusted so that it was proportional to the relationship, cos^2^*θ*. The CSDA range in silica dioxide (SiO_2_) published by the Particle Data Group^25^ was utilized. For the HS topographic map, the digital elevation map (DEM) published by the Geospatial Information Authority of Japan, Ministry of Land, Infrastructure, Transport and Tourism (GSI) was utilized. Accuracy of the contour position was less than ±5 m. The path lengths from Mu-A were calculated based on the GSI’s topographic map with an elevation and azimuth angular interval of 1° and the transmitted muon flux was calculated for each path. The derived flux was integrated according to the geometrical acceptance of the tracker to calculate the expected muon counts in each bin before and after passing through HS.

## Additional Information

**How to cite this article**: Tanaka, H. K. M. Instant snapshot of the internal structure of Unzen lava dome, Japan with airborne muography. *Sci. Rep.* **6**, 39741; doi: 10.1038/srep39741 (2016).

**Publisher's note:** Springer Nature remains neutral with regard to jurisdictional claims in published maps and institutional affiliations.

## Supplementary Material

Supplementary Movie 1

Supplementary Movie 2

Supplementary Video Legends

## Figures and Tables

**Table 1:** Normalized number of open-sky muon events.

Elevation angle range (deg)	Azimuth angle range (deg)				
	−41 ± 8°	−24 ± 12°	0 ± 12°	24 ± 12°	41 ± 8°
60 ± 3°	31.14	47.63	69.64	54.04	40.82
53 ± 4°	46.65	95.86	150.3	120.8	63.53
41 ± 8°	71.28	171.7	272.1	232.5	97.63
24 ± 12°	83.86	173.3	346.3	255.1	101.7

**Table 2:** Normalized number of transmitted muon events.

Elevation angle range (mrad)	Azimuth angle range (mrad)				
	−41 ± 8°	−24 ± 12°	0 ± 12°	24 ± 12°	41 ± 8°
60 ± 3°	30.95	48.02	58.78	52.41	33.22
53 ± 4°	48.32	91.70	132.7	105.5	62.14
41 ± 8°	71.76	157.3	242.6	227.2	94.62
24 ± 12°	72.74	121.1	195.1	176.5	104.8

**Table 3:** Muon transmission rate.

Elevation angle range (mrad)	Azimuth angle range (mrad)				
	−41 ± 8°	−24 ± 12°	0 ± 12°	24 ± 12°	41 ± 8°
60 ± 3°	0.994	1.008	0.844	0.970	0.814
53 ± 4°	1.036	0.957	0.883	0.873	0.978
41 ± 8°	1.007	0.917	0.892	0.977	0.969
24 ± 12°	0.868	0.699	0.563	0.692	1.030
