# Supplementary material for: Instant snapshot of the internal structure of Unzen lava dome, Japan with airborne muography
Source: Sci Rep. 2016 Dec 23;6:39741. doi: 10.1038/srep39741 (PMC5180201; doi:10.1038/srep39741)
Supplement: Supplementary Video Legends [file srep39741-s3.pdf]

Instant snapshot of the internal structure of Unzen lava dome, Japan with airborne muography

Hiroyuki K.M. Tanaka[1]

Earthquake Research Institute, The University of Tokyo, 1-1-1 Yayoi, Bunkyo, Tokyo 113-0032, Japan

*Correspondence to ht@riken.jp

Supplementary Movie 1. Apparatus flying to the lava dome. The muon trackers were equipped inside the aircraft that flew and hovered near the peak region of Heisei-Shinzan lava dome of Unzen volcano, Japan to record muons.

Supplementary Movie 2. Aircraft Hovering. The aircraft hovered at a position of 200 m southwest from the peak of the 1994 spine at an altitude of 1350 m above sea level (asl) for 157 minutes in total. The location of the aircraft was continuously monitored.
